# Liprin-α-Mediated Assemblies and Their Roles in Synapse Formation

**DOI:** 10.3389/fcell.2021.653381

**Published:** 2021-03-19

**Authors:** Xingqiao Xie, Mingfu Liang, Cong Yu, Zhiyi Wei

**Affiliations:** ^1^Department of Biology, Southern University of Science and Technology, Shenzhen, China; ^2^Academy for Advanced Interdisciplinary Studies, Southern University of Science and Technology, Shenzhen, China; ^3^Guangdong Provincial Key Laboratory of Cell Microenvironment and Disease Research, Shenzhen Key Laboratory of Cell Microenvironment, Shenzhen, China; ^4^Brain Research Center, Southern University of Science and Technology, Shenzhen, China

**Keywords:** SYD2, scaffold protein, presynaptic active zone, LLPS, protein structure, coiled coil, protein–protein interaction

## Abstract

Brain’s functions, such as memory and learning, rely on synapses that are highly specialized cellular junctions connecting neurons. Functional synapses orchestrate the assembly of ion channels, receptors, enzymes, and scaffold proteins in both pre- and post-synapse. Liprin-α proteins are master scaffolds in synapses and coordinate various synaptic proteins to assemble large protein complexes. The functions of liprin-αs in synapse formation have been largely uncovered by genetic studies in diverse model systems. Recently, emerging structural and biochemical studies on liprin-α proteins and their binding partners begin to unveil the molecular basis of the synaptic assembly. This review summarizes the recent structural findings on liprin-αs, proposes the assembly mechanism of liprin-α-mediated complexes, and discusses the liprin-α-organized assemblies in the regulation of synapse formation and function.

## Introduction

In the brain, neurons are connected and communicated with each other via highly specialized intercellular junctions, termed synapses. Chemical synapses are enriched with numerous proteins, including ion channels, receptors, enzymes, and scaffold proteins. These synaptic proteins are spatiotemporally orchestrated to control the release and receiving of neurotransmitter at the presynaptic and postsynaptic sites respectively to transmit neuronal signals (Broadie and Richmond, 2002; Sudhof, 2008, 2018; Chua et al., 2010; Harris and Weinberg, 2012; Missler et al., 2012; Ackermann et al., 2015). The precise signal response and transduction in synapse depend on the proper assemblies of presynaptic active zone and postsynaptic density, in which many scaffold proteins play essential roles to organize these assemblies through protein-protein interactions (Schoch and Gundelfinger, 2006; Feng and Zhang, 2009; Haucke et al., 2011; Sheng and Kim, 2011; Sudhof, 2012; Petzoldt et al., 2016; Biederer et al., 2017; Torres and Inestrosa, 2018; Zeng et al., 2018; Gramlich and Klyachko, 2019).

Liprin-α family proteins are core synaptic scaffolds and important for the assembly and maturation of synapses. By interacting with various synaptic proteins, liprin-αs participate in both presynaptic and postsynaptic functions, including active zone assembly, neurotransmitter release, and synaptic cargo transport (Zhen and Jin, 2004; Spangler and Hoogenraad, 2007; Stryker and Johnson, 2007; Sigrist, 2009; Ackermann et al., 2015; Wong et al., 2018) (Table 1). The liprin-α family contains four members (liprin-α1/2/3/4) in vertebrates and one member each in *C. elegans* and *Drosophila*, named SYD-2 and Dliprin-α, respectively (Serra-Pages et al., 1998; Zhen and Jin, 1999; Kaufmann et al., 2002; Astigarraga et al., 2010). In mammals, while liprin-α1 is ubiquitously expressed, liprin-α2/3 are mainly expressed in the brain and liprin-α4 was found in both the brain and testis (Serra-Pages et al., 1998; Zurner and Schoch, 2009; Wong et al., 2018). Liprin-α2/3 proteins were shown to have both presynaptic and postsynaptic localization at excitatory synapses in both hippocampus and cultured neurons (Spangler et al., 2011; Zurner et al., 2011), indicating that liprin-αs have separate presynaptic and postsynaptic functions. The dysfunction or depletion of liprin-αs in worms and mice led to abnormal ultrastructure of the active zone and impaired synaptic transmission (Zhen and Jin, 1999; Patel et al., 2006; Kittelmann et al., 2013; Spangler et al., 2013; Wong et al., 2018). Notably, mammalian liprin-α1 was extensively characterized in non-neuronal cells by its functions in cell motility (de Curtis, 2011). Considering that liprin-α1 is the predominant liprin-α isoform in glial cells (Spangler et al., 2011), liprin-α1 may also contribute to the synapse development through glial-neuron interactions.

**TABLE 1:** Interactions meditated by liprin-α family members.

Interactor	Liprin-α family member	Interaction region	Cellular function	References
LAR, PTPδ, PTPσ Dlar PTP-3	α1, 2, 3, 4 Dliprin-α SYD2	SAM123	Synaptogenesis, neuron development, acrosome reaction	Serrapages et al., 1995; Serra-Pages et al., 1998; Kaufmann et al., 2002; Wyszynski et al., 2002; Ackley et al., 2005; Dunah et al., 2005; Astigarraga et al., 2010; Kiok et al., 2011; Joshi et al., 2014; Bomkamp et al., 2019; Wakita et al., 2020; Xie et al., 2020
CASK	α1, 2, 3, 4	SAM123	Neurotransmitter release	Olsen et al., 2005; Samuels et al., 2007; Wei et al., 2011; LaConte et al., 2016; Wu et al., 2016
Liprin-β1	α1, 2, 3 Dliprin-α SYD2	SAM123	Synaptogenesis, cell motility	Serra-Pages et al., 1998; Astigarraga et al., 2010; Wei et al., 2011; Chiaretti et al., 2016
mSYD1A SYD1	α2 SYD2	SAM123	Active zone formation	Chia et al., 2012; Owald et al., 2012; Wentzel et al., 2013; McDonald et al., 2020
RSY-1	SYD2	SAM123	Regulation of presynaptic assembly	Patel and Shen, 2009; Chia et al., 2013
CAMKIIα	α1	SAM123	Synapse morphogenesis	Hoogenraad et al., 2007
Unc13B	Dliprin-α	Coiled-coil region	Active zone formation	Bohme et al., 2016
Liprin-γ	Dliprin-α	Coiled-coil region	Synaptogenesis	Astigarraga et al., 2010
KIF1A Kinesin-3	α1,2, Dliprin-α SYD2	Coiled-coil region	Synaptic vesicles transport	Shin et al., 2003; Wagner et al., 2009; Stucchi et al., 2018
Tanc2	α2	Coiled-coil region	Postsynaptic development	Stucchi et al., 2018
Liprin-α	α1, 2, 3, 4 SYD2	Coiled-coil region	Presynaptic formation	Serra-Pages et al., 1998; Taru and Jin, 2011; Astro et al., 2016
RIM1	α3, 4	CC2	Neurotransmitter release	Schoch et al., 2002
ELKS, CAST	α1,2,3,4 SYD2	CC2	Active zone formation	Ko et al., 2003b; Dai et al., 2006; Kittelmann et al., 2013
GIT1	α1, 2, 3, 4 Dliprin-α SYD2	SAH	AMPA receptor targeting, cell spreading	Ko et al., 2003a, b; Totaro et al., 2007; Asperti et al., 2011; Liang et al., 2019; McDonald et al., 2020
mDia	α1, 3	LCR	Stress fiber formation	Sakamoto et al., 2012; Brenig et al., 2015
GRIP1	α1, 2, 3, 4	PBM	AMPA receptor targeting	Wyszynski et al., 2002; Im et al., 2003; Zurner and Schoch, 2009; Chatterjee and Roy, 2017
LNX1	α1,2,3	PBM	Ubiquitination of liprin-α	Lenihan et al., 2017a, b
PP2A B56γ	α1 Dliprin-α	PBBM	Regulation of synaptic materials in distal axons	Arroyo et al., 2008; Li et al., 2014
PP2Aα	α1	Unknown	Trafficking to the ciliary tip, Hedgehog signaling	Liu et al., 2014
Kinesin-1	Dliprin-α	Unknown	Synaptic vesicles transport	Miller et al., 2005
KIF7	α1	Unknown	Trafficking to the ciliary tip, Hedgehog signaling	Liu et al., 2014
ING4	α1	Unknown	Cell growth and motility	Unoki et al., 2006; Shen et al., 2007
α-Dystrobrevin-1	α1	Unknown	Neuromuscular junction development	Gingras et al., 2016; Bernadzki et al., 2017
PSD95	α1	Unknown	Postsynaptic organization	Huang et al., 2017
CDK5	α1	Unknown	Postsynaptic organization	Huang et al., 2017
PARP1	α1	Unknown	P65 transcriptional activation	Gu et al., 2019
EphA2	α1	Unknown	Cell motility	Buraschi et al., 2020

**FIGURE 1:**
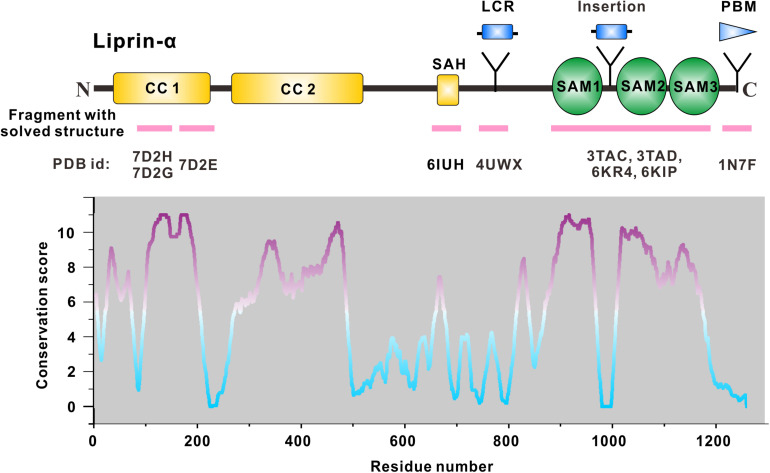
Domain organization of liprin-α. The diagram depicts the domains or regions of liprin-α. CC, coiled-coil region; SAH, single alpha helix; SAM, sterile-α-motif domain. Isoform or species-specific regions were shown above the schematic diagram. LCR, a liprinα3 core region in liprin-α3; Insertion, an inserted loop region in liprin-α2/3/4; PBM, a PDZ binding motif in vertebrate liprin-αs. Regions of liprin-α with solved structure were highlighted by pink lines under the diagram. PDB ids were shown under the pink lines (7D2H and 7D2G for liprin-α2_H2, 7D2E for liprin-α2_H3; 6IUH for the liprin-α2_SAH/GIT1_PBD complex; 4UWX for the liprin-α3_LCR/mDia_DID complex; 3TAC, 3TAD, 6KR4, and 6KIP for the SAM123 structures in complex with CASK_CaMK, liprin-β1_SAM123, LAR_D1D2, and PTPδ_D2, respectively; 1N7F for the liprin-α1_PBM/GRIP1_PDZ6 complex). The value curve at the bottom panel indicates sequence conservation of liprin-α proteins. The conservation score for each residue was calculated in Jalview (Waterhouse et al., 2009) using the sequence alignment of liprin-α family members across species, including *Caenorhabditis elegans, Drosophila melanogaster, Danio rerio, Xenopus tropicalis, Gallus gallus, Mus musculus*, and *Homo sapiens*. The scores from 0 to 11 indicate the most variable to the most conserved state of each residue, colored from cyan to purple gradually.

The sequence analysis shows that liprin-α proteins share an evolutionarily conserved domain organization, characterized by N-terminal coiled coils and C-terminal three tandem SAM (sterile-α-motif) domains (SAM123), which are the known regions for protein binding (Spangler and Hoogenraad, 2007) (Figure 1 and Table 1). The similar domain organization was found in other liprin-type scaffold proteins, liprin-β1/2 and liprin-γ (Serra-Pages et al., 1998; Astigarraga et al., 2010). In addition, some isoform/species-specific regions found in liprin-αs endow additional interactions and functions (Figure 1). In this review, we focus on the synaptic assemblies that are organized and regulated by liprin-αs. To approach this topic, we describe the high-resolution structures of liprin-αs and their complexes, dissect the protein–protein interactions in these structures, and discuss the potential implications of these structural findings on the regulation of protein assemblies required for synaptogenesis and synaptic functions.

## The C-Terminal Sam123: Providing Multiple Protein-Binding Surfaces for Supramolecular Assemblies

Liprin-α was first identified as the binding protein of leukocyte common antigen-related receptor protein tyrosine phosphatases (LAR-RPTPs) that are cell surface receptors containing three members, LAR, PTPδ and PTPσ (Serrapages et al., 1995; Um and Ko, 2013; Han et al., 2016). The liprin-α/LAR-RPTP interaction is important for synaptogenesis as indicated by *in vitro* heterologous synapse-formation assays and *in vivo* characterizations of excitatory synaptic morphogenesis and electrophysiological function (Kaufmann et al., 2002; Dunah et al., 2005; Um and Ko, 2013; Han et al., 2018, 2019, 2020; Won and Kim, 2018). Biochemical analysis determined binding regions at the three SAM domains of liprin-α (also referred to as liprin homology domain or LHD) and the cytoplasmic phosphatase domains of LAR-RPTPs (Serra-Pages et al., 1998). The SAM domain is one of the most abundant protein-binding domains and consists of ∼70 amino acids folded as a five-helix bundle (Qiao and Bowie, 2005; Denay et al., 2017). Although the SAM domain appears once in most SAM-containing proteins, all liprin proteins contain three tandem SAM domains. To date, the SAM123 region of liprin-αs have been reported to mediate a diverse array of interactions with kinases (CASK and CAMKII), phosphatases (LAR-RPTPs), and other scaffolds (liprin-β1/2, mSYD1 and RSY-1) in spine formation and presynaptic assembly (Kaufmann et al., 2002; Olsen et al., 2005; Hoogenraad et al., 2007; Patel and Shen, 2009; Wentzel et al., 2013) (Table 1).

**FIGURE 2:**
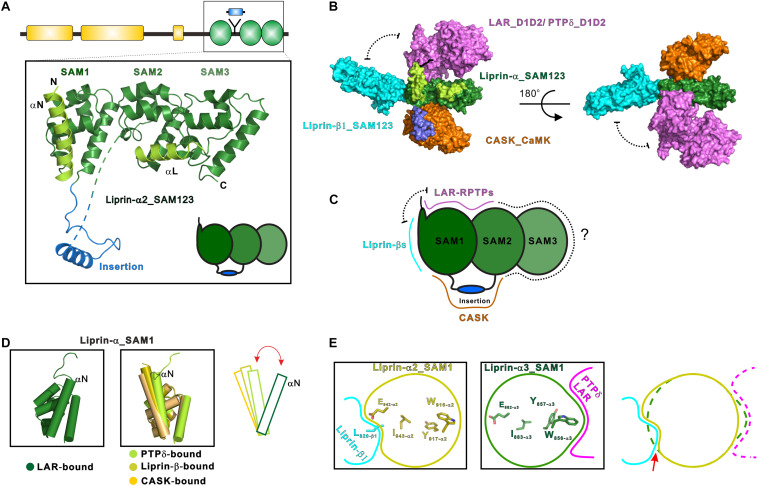
Liprin-α SAM123 mediates supramolecular assemblies. **(A)** The structure of liprin-α SAM123. The SAM123 structure is derived from the liprin-α2/CASK complex (PDB id: 3TAC). **(B)** A structure model showing the SAM123-mediated protein assembly. The SAM123 structures in complex with CASK (PDB id: 3TAC), liprin-β1 (3TAD), and LAR (6KR4) are superimposed. **(C)** A schematic model indicates the target-binding surfaces of the liprin-α SAM123. Some surface patches on SAM2 and SAM3 may be also involved in protein binding. **(D)** Conformational dynamics of the αN-helix in the SAM1 domain. The rotational change of αN was indicated by a red bidirectional arrow. **(E)** Allosteric regulation of the binding of the liprin-α SAM123 to liprin-β1 and LAR-RPTPs. The SAM1 residues that are involved in the allosteric regulation were shown as sticks. The conformational propagation of these SAM1 residues upon LAR-RPTP binding leads to the clash (indicated by a red arrow) between the liprin-α SAM1 and liprin-β1 and thus blocks the liprin-α/β interaction.

The first structural characterization of SAM123 in liprin-α2 revealed that the three SAM domains are integrated together as a structural module (Wei et al., 2011) (Figure 2A). This arrangement of the SAM domains creates several protein-binding surfaces across the SAM domains. Specifically, SAM123 was found to interact with the CaM kinase domain (CaMK) of CASK and SAM123 of liprin-β1 simultaneously, indicating that SAM123 mediates protein assemblies by using different interfaces (Figures 2B,C). This structural indication was further supported by two recent structural studies of liprin-α3 in complexes with the cytoplasmic phosphatase domains of two LAR-RPTP proteins, LAR and PTPδ (Wakita et al., 2020; Xie et al., 2020). The binding surface for LAR-RPTPs on SAM123 shows no overlap with that for either CASK or liprin-β (Figures 2B,C). Consistent with these structural findings, two ternary complexes of CASK/liprin-α2/liprin-β1 and CASK/liprin-α3/LAR mediated by SAM123 were formed in solution. To further dissect the reported protein-binding modes for SAM123, we analyzed each structural element in SAM123 contributing to the protein–protein interaction.

### SAM1

Consistent with its high sequence conservation (Figure 1), the SAM1 domain is crucial for the SAM123-mediated liprin-α complexes with liprin-βs, LAR-RPTPs, and CASK (Figure 2B). The crystal structure of the liprin-α2_SAM123/liprin-β1_SAM123 complex and *in vitro* binding assays reveal that the binding of the liprin-α SAM1 domain to the liprin-β SAM3 domain is through the typical SAM/SAM interaction (Wei et al., 2011), which has been found in many SAM-containing proteins for homo-oligomerization (Qiao and Bowie, 2005). Although liprin-βs were much less studied than liprin-αs, the formation of the liprin-α/liprin-β heterodimer that involves the six SAM domains enlarges accessible surfaces on the SAM domains for protein binding and that extends the capacity of liprin-αs to assemble more supramolecular complexes. Indeed, *Drosophila* liprin-α and liprin-β were reported to interact with each other via their SAM domains and function together for normal synapse formation (Astigarraga et al., 2010). Compared with the other two SAM domains, SAM1 in liprin-α contains an additional helix (αN) at the N terminus (Figure 2A). The αN-helix in the LAR-bound liprin-α3_SAM123 structure shows a large rotation (Figure 2D). This rotational change of the αN-helix alters the available surface on the SAM1 domain, implying a regulation mechanism for the binding of an unknown partner to SAM1. Of note, the αN rotation is unlikely to be induced by its binding to LAR-RPTPs, as the αN-helix in the PTPδ-bound SAM1 domain does not show a similar rotation (Figure 2D).

Interestingly, despite that the LAR- and liprin-β1-binding surfaces on the SAM1 domain are not overlapped, the binding of liprin-α to LAR and to liprin-β1 is mutually exclusive (Xie et al., 2020). Structural comparison of the SAM1 structures bound to LAR-RPTPs and liprin-β1 shows that the LAR binding to liprin-α triggers a conformational propagation of several interacting residues in the SAM1 domain and leads to the steric inhibition of liprin-β1 binding (Figure 2E). Considering that the multiple roles of liprin-αs in the synapse, this allosteric regulation is likely to control the varied components in different protein assemblies mediated by liprin-αs.

### SAM2

The liprin-α SAM2 domain is sandwiched by SAM1 and SAM3, which bury the typical protein-binding surfaces of the SAM2 domain. On the other hand, SAM1 and SAM2 together generate new binding surfaces for LAR-RPTPs and CASK (Figures 2B,C). Specifically, the phosphatase domains of LAR and PTPδ interact with a cleft between the two SAM domains, whereas a surface patch opposite to the cleft facilitates the binding of CASK to liprin-α.

### SAM3

The liprin-α SAM3 domain has not been found to participate in the protein-target recognition. Considering that the SAM123-mediated interactions are not fully explored and the domain organization in liprin-αs requires the three SAM domains together, we propose that the SAM3 domain may also contribute its surface for the interactions with certain binding partners of liprin-αs (Figure 2C). Of note, although the liprin-β SAM3 binds to the liprin-α SAM1, the liprin-α SAM3 cannot interact with the liprin-α SAM1 to form oligomers, as the interface residues in the liprin-β SAM3 are not conserved in the liprin-α SAM3 (Wei et al., 2011).

### Accessory Elements

In addition to the SAM domains, some accessory elements in the SAM123 region are required for the complex formation between liprin-α and its specific binding partners (Figures 1, 2A). In both the crystal structures of liprin-α3_SAM123 in complex with the two tandem phosphatase domains (D1D2) of LAR and with the second phosphatase domain (D2) of PTPδ, a loop at the N-terminal to the αN-helix was found to provide a second binding site for the D2 phosphatase domains of LAR and PTPδ, suggesting a conserved two-site binding mode between liprin-αs and LAR-RPTPs (Figure 2B) (Wakita et al., 2020; Xie et al., 2020).

The liprin-α2/CASK interaction involves an insertion region between the SAM1 and SAM2 domains (Figures 2A,B), in which a valine-tryptophan-valine (“VWV”) motif is buried in a hydrophobic pocket on the CaMK domain of CASK (Wei et al., 2011). This insertion is conserved in neuronal specific liprin-αs, liprin-α2/3/4, but not in liprin-α1 and invertebrate liprin-αs. Notably, the insertion of human liprin-α2 is encoded by two exons (Zurner and Schoch, 2009), suggesting that the binding of CASK to liprin-α is an evolutionary gain and is regulated by alternative splicing.

### Regulation Mechanisms of the SAM123-Mediated Interactions

The structural and biochemical characterizations of the SAM123-mediated interactions provide mechanistic insights into the understanding of liprin-α’s functions. Structure-guided mutagenesis study suggested that several X-linked mental retardation-associated mutations of CASK impair the binding of CASK to liprin-α2 (Najm et al., 2008; Tarpey et al., 2009; Wei et al., 2011). Because the VWV motif in the insertion of liprin-α2 is critical for the liprin-α/CASK interaction, Mint-1 and Caskin, which also bind to CASK using the similar VWV motif (Stafford et al., 2011; Wu et al., 2020), may interfere with this interaction. As the Veli/CASK/Mint-1 tripartite complex is involved in neurotransmitter release (Olsen et al., 2005), the CASK-binding competition between liprin-αs and Mint-1 is likely a regulation mechanism for the distribution and composition of the presynaptic assemblies.

The highly conserved association between liprin-αs and LAR-RPTPs has been extensively characterized in the synapse initiation, assembly and maintenance (Kaufmann et al., 2002; Ackley et al., 2005; Dunah et al., 2005; Han et al., 2018; Ozel et al., 2019). Catalytically inactive mutants of LAR-RPTPs failed to control axon growth or synaptogenesis (Johnson et al., 2001; Dunah et al., 2005). The SAM123 regions of liprin-αs bind to the catalytically inactive D2 domains, instead of the active D1 domains of LAR-RPTPs (Serra-Pages et al., 1998; Astigarraga et al., 2010). However, the structural findings on the liprin-α3/LAR complex implied that by binding to liprin-α proteins, LAR forms clusters on the cell surface, which promotes the self-association of the LAR D1 domain, blocking its substrate binding (Xie et al., 2020). Thus, liprin-αs may regulate the synapse formation by attenuating LAR’s activity via forming the large protein assembly. Importantly, the N-terminal coiled-coil regions of liprin-αs are also required for the cluster formation of the liprin-α/LAR complex by oligomerizing liprin-αs, which will be discussed in the next section.

## The N-Terminal Coiled Coils: The Self-Assembly of Liprin-α

The N-terminal coiled coils are essential for synaptic functions of liprin-αs. In *C. elagans*, the coiled-coil region of SYD2 is necessary and sufficient to suppress synaptic defects caused by a loss-of-function mutant of SYD2 (Dai et al., 2006; Taru and Jin, 2011) and to assemble functional synapses (Chia et al., 2013). The N-terminal conserved region of liprin-α contains three predicted coiled-coil segments, in which the first two segments are named CC1 and CC2 (Figure 1). The third one was recently characterized as a single α-helix (SAH) in solution (Liang et al., 2019), suggesting that only two coiled coils exist in liprin-α. The N-terminal region was suggested to organize self-assembly of liprin-αs (Serra-Pages et al., 1998; Astigarraga et al., 2010; Taru and Jin, 2011), presumably mediated by the coiled-coil formation despite lacking detailed investigations. In addition, several synaptic proteins (e.g., RIM, ELKS, GIT1, KIF1A, and TANC2) interact with liprin-αs via this coiled-coil region (Schoch et al., 2002; Ko et al., 2003b; Shin et al., 2003; Stucchi et al., 2018) (Table 1).

### CC1

CC1 is the most conserved region among vertebrate liprin-αs and *C. elegans* SYD2 (Figure 1). In *C. elegans*, SYD2 functions together with SYD1, both of which are required for the synapse formation of hermaphrodite specific neurons (Dai et al., 2006). In mouse model, the depletion of liprin-α2, liprin-α3, or mSYD1A (a mammalian homolog of SYD1) led to the similar synaptic defects, including decreased vesicle docking and impaired synaptic transmission (Spangler et al., 2013; Wentzel et al., 2013; Wong et al., 2018). Interestingly, a single substitution of R184 with cysteine in the CC1 region of SYD2 was genetically identified as a gain-of-function mutation, which promote synaptic assembly even in the absence of SYD1 (Dai et al., 2006). An electron microscopic study demonstrated that the worm bearing the R184C mutation had highly enhanced protein-dense matrix at the presynaptic active zone (Kittelmann et al., 2013), suggesting the promoted active zone formation. Biochemical analysis indicated that the R184C mutation promotes the oligomerization of SYD2 (Taru and Jin, 2011; Kittelmann et al., 2013; Liang et al., 2020).

**FIGURE 3:**
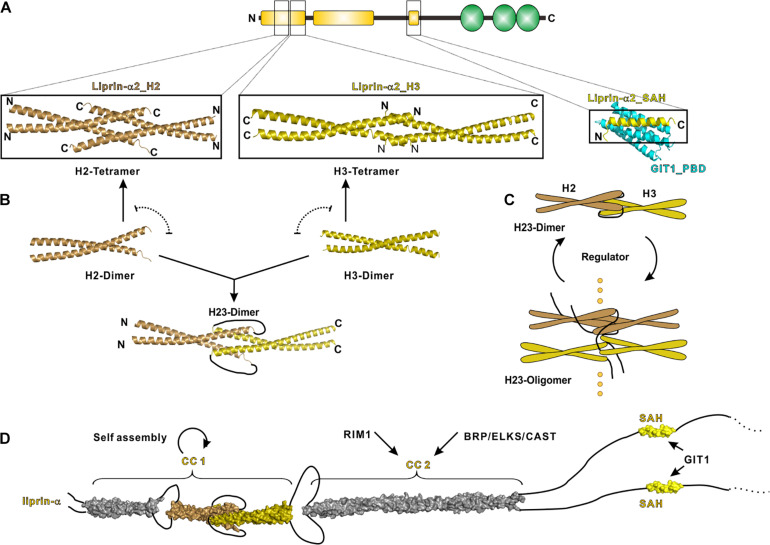
N-terminal coiled coils mediate the self-assembly of liprin-α. **(A)** The solved structures of liprin-α N-terminus were indicated by black boxes. The H2 (PDB id: 7D2H), H3 (7D2E), and SAH (6IUH) segment structures of liprin-α2 were shown. **(B)** A model indicating that the dimeric H2 and H3 competitively transit between homo-tetramers and a H23-dimer. **(C)** A schematic cartoon showing the self-assembly mechanism of liprin-α CC1. The transition between dimers and higher oligomers may be controlled by unknown regulators. **(D)** A structure model showing the complete N-terminal structure of liprin-α. The H1 segment and CC2 was modeled as coiled coils and shown in gray.

CC1 contains three α-helices termed H1, H2 and H3, respectively, connected by short loops. Recently, crystal structures of H2 and H3 in liprin-α2 were determined, which reveal the molecular mechanism underlying the self-assembly of liprin-αs (Liang et al., 2020). In these structures, the H2- and H3-helices are both homo-tetramerized yet through different assembly modes (Figure 3A). Combined with extensive biochemical characterizations, these structures suggest an assembly model of CC1, in which the dimeric coiled coils of H2 and H3 are either interacted with each other to form a H23 dimer or self-associated to form tetramers (Figure 3B). The gain-of-function mutation in CC1 diminishes the H2/H3 interaction and inhibits the H23 dimer formation, in return promoting H2 and H3 tetramerization and then CC1 oligomerization (Liang et al., 2020) (Figure 3C). Therefore, the mutation-promoted self-assembly of liprin-α/SYD2 provides multiple protein-binding sites and thus enhances the presynaptic recruitment of other synaptic proteins, such as ELKS for the active zone formation (Dai et al., 2006; Kittelmann et al., 2013).

This gain-of-function effect implies that the assembly of liprin-αs under the physiological condition is determined by unknown regulator(s). The regulator(s) may increase the self-assembly of liprin-αs through stabilizing the H23 dimer or vice versa (Figure 3C). SYD1 is a promising positive regulator in this regard, as the genetic analysis indicated that SYD1 acts as an upstream factor required for SYD2’s activity on synaptogenesis (Dai et al., 2006). The coiled-coil region in SYD2 was suggested to associate with SAM123 to adopt an auto-inhibition conformation (Patel and Shen, 2009), presumably locking the H23 dimer to prevent the self-assembly of SYD2. As the mammalian homolog of SYD1 was reported to interact with liprin-α SAM123 (Wentzel et al., 2013), it is tempting to speculate that the SYD1 binding releases the auto-inhibition of liprin-α or SYD2 and thereby leads to the promoted self-assembly. The potential negative regulator is RSY-1 that inhibits the synapse formation in *C. elegans* (Patel and Shen, 2009). RSY-1 was found to associate with SYD2 and antagonize the pro-synaptic function of SYD2 (Patel and Shen, 2009).

### CC2

The liprin-α CC2 is the binding region for ELKS and RIM, both of which participate in the ultrastructure organization and neurotransmitter release at the active zone (Ohtsuka et al., 2002; Schoch et al., 2002; Kittel et al., 2006; Wang et al., 2009, 2016; Held et al., 2016) (Figure 3D). Recent studies showed that liprin-α proteins undergo either phosphorylation-dependent phase transition or co-phase separation with ELKS (Emperador-Melero et al., 2020; Liang et al., 2020; McDonald et al., 2020). These findings indicate that the self-assembled liprin-αs act as a hub to dynamically recruit ELKS, RIM, and other binding partners to form the electron-dense protein aggregates in the presynaptic active zone observed by electron microscopy (Fouquet et al., 2009; Kittelmann et al., 2013; Spangler et al., 2013).

In addition to the liprin-α-mediated phase separation, RIM and RIM-binding protein (RIM-BP) co-phase separate and cluster calcium channels to form the active zone-like condensate *in vitro* (Wu et al., 2019). It is likely that the interplay between the two types of condensates, the liprin-α/ELKS condensate and the RIM/RIM-BP condensate, in the active zone contributes to the highly patterned distributions of the active zone proteins (Emperador-Melero and Kaeser, 2020). Consistently, the purified RIM and calcium channel proteins are differentially distributed in the two condensed phases in the presence of the self-assembled liprin-α (Liang et al., 2020), suggesting that liprin-α serves as a molecular sieve in protein condensates to facilitate the compartmentalization of synaptic proteins in the active zone.

### Single α-Helix

The SAH region of liprin-α contains a leucine/aspartate (LD)-like motif that binds to the C-terminal PBD domain of GIT1 (Ko et al., 2003a; Asperti et al., 2011). GIT1 is a GTPase-activating protein (GAP) that plays regulatory roles in neurotransmitter release and spine formation in mice (Zhang et al., 2003, 2005; Podufall et al., 2014; Hong and Mah, 2015). This liprin-α/GIT1 interaction was defined at the postsynapse specifically for AMPA receptor clustering (Wyszynski et al., 2002; Im et al., 2003). The crystal structure of the SAH/PBD complex reveals that the SAH interacts with the GIT1_PBD through a mode differing from the canonical LD binding mode (Liang et al., 2019) (Figure 3A). As the PBD domain of GIT1 interacts with other LD-containing proteins (Schmalzigaug et al., 2007; Zhang et al., 2008), the structural finding reveals how GIT1 specifically recognized liprin-α through the SAH-mediated interaction in the synapse.

Although liprin-αs and GIT1 are also enriched at the presynaptic site, the presynaptic function of the liprin-α/GIT1 complex remain unknown. In addition, because GIT1 interacts with Stonin2 and Piccolo for synaptic vesicle recycling (Kim et al., 2003; Podufall et al., 2014) and liprin-α regulates docking and exocytosis of synaptic vesicles (Wong et al., 2018), the liprin-α/GIT1 interaction may link the cycling synaptic vesicles to the presynaptic active zone.

## Other Protein-Binding Regions in Liprin-αs

### Liprin-α3 Core Region (LCR)

**FIGURE 4:**
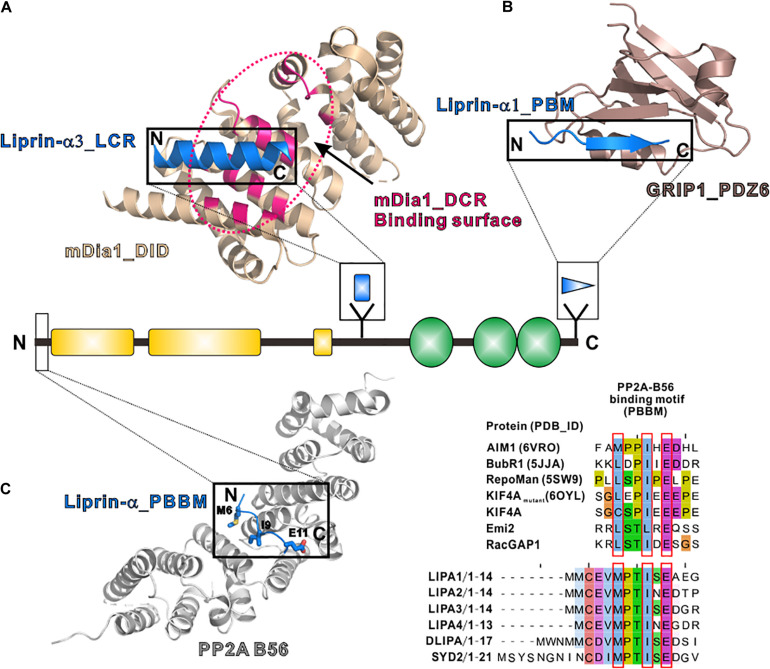
Additional regions of liprin-α in protein–protein interactions. **(A)** The structure of the LCR in complex with mDia (PDB id: 4UWX). Base on another solved structure of mDia (PDB id: 2BAP), the surface on mDia that is involved in the autoinhibition formation was highlighted by a dotted hotpink circle, showing the two binding sites are largely overlapped. **(B)** The structure of the PBM in complex with the PDZ6 domain of GRIP1 (PDB id: 1N7F). **(C)** The manually modeled structure of the PP2A-B56/liprin-α_PBBM complex. The sequences of the PBBMs from different proteins in the solved structures were aligned with the PBBMs of liprin-αs. The residues that are involved in B56 binding were highlighted by red boxes.

The LCR, only found in liprin-α3, folds as a short α-helix and interacts with mDia, an actin nucleator, to regulate the dynamics of actin filaments (Sakamoto et al., 2012; Brenig et al., 2015). The structural study of the LCR/mDia complex showed that the LCR prevents mDia from adopting an auto-inhibited conformation (Figure 4A), therefore promoting actin polymerization in the cell (Brenig et al., 2015). Although the LCR sequence is not conserved in other liprin-α proteins, liprin-α1 was found to interact with mDia (Sakamoto et al., 2012). Whether liprin-α1 binds to mDia by using a similar sequence in other regions or a different sequence need further study.

### PDZ Binding Motif (PBM)

PDZ binding motifs (PBMs) were found in the C-termini of vertebrate liprin-αs (Im et al., 2003; Zurner and Schoch, 2009) but not in their invertebrate homologs. The sixth PDZ domain of GRIP1 was identified to bind to the PBM of liprin-α (Wyszynski et al., 2002). The PBM-binding pocket of the dimeric GRIP1_PDZ6 is located at the distal sides of the dimer interface (Figure 4B). Dimeric PDZ6 domain mediates GRIP1 multimerization, which enhances the binding of the neighboring PDZ5 domain to receptors, clusters Glutamate receptor 2/3, and regulates AMPA receptor targeting in murine postsynapse (Wyszynski et al., 2002; Ko et al., 2003a). Alternative RNA splicing may generate some liprin-α protein products without the PBMs (Zurner and Schoch, 2009) and thus regulate the receptor binding for liprin-αs. As PBMs do not present in SYD2 or Dliprin-α, the PBM-mediated postsynaptic interaction is likely an evolutionary gain for vertebrate liprin-αs.

### PP2A-B56 Binding Motif (PBBM)

In addition to tyrosine phosphatases, liprin-αs were found to interact with a serine/threonine phosphatase, protein phosphatase 2A (PP2A) (Arroyo et al., 2008; Li et al., 2014; Liu et al., 2014). Drosophila liprin-α, SYD1 and PP2A form a linear pathway for the presynapse formation (Li et al., 2014). In mammals, liprin-αs function together with PP2A in controlling the phosphorylation level of KIF7 and activating Hedgehog-target genes (Arroyo et al., 2008; Liu et al., 2014). The binding of liprin-α to the PP2A holoenzyme was mapped to the regulatory subunit of PP2A, B56 (Arroyo et al., 2008; Li et al., 2014). A systematic study of the PP2A-B56 binding motif (PBBM) uncovers a L/M-X-X-I-X-E consensus sequence motif (Hertz et al., 2016), which is also found in the very N-terminal parts of liprin-αs across different species (Figure 4C). Based on a previously solved structure of the B56/PBBM complex, the B56/liprin-α complex structure was modeled, showing that the PBBM sequence of liprin-α fits well to the typical PBBM-binding pocket of B56 (Figure 4C). Notably, as the phosphorylation of a threonine residue in the PBBM of Emi2 or RacGAP1 promotes their binding to B56 (Hertz et al., 2016), the strictly conserved threonine in the PBBM of liprin-α may be phosphorylated to regulate the liprin-α/PP2A interaction (Figure 4C).

## Conclusion and Perspectives

**FIGURE 5:**
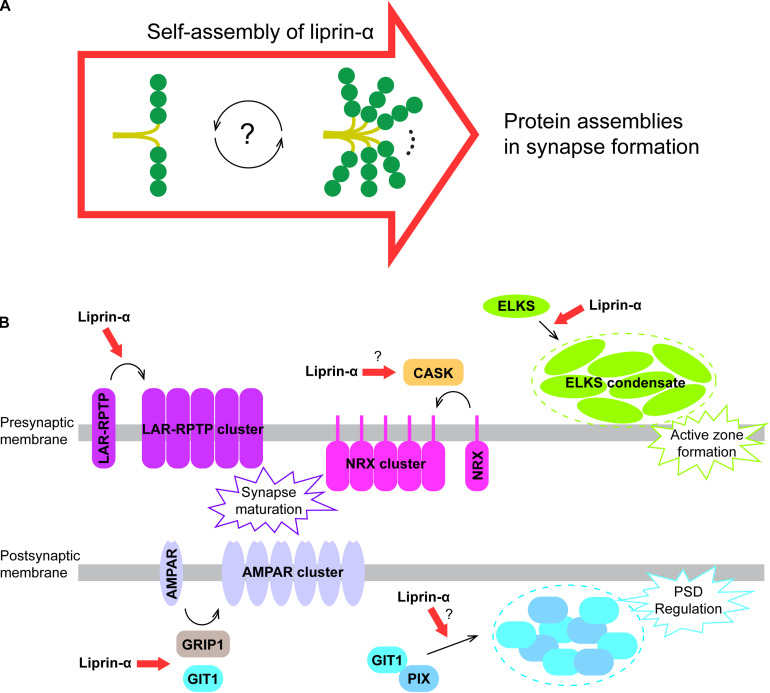
A proposed model for liprin-α assembly in mediating protein accumulation in synapse development. **(A)** Liprin-α is self-assembled to form a large complex and to mediate protein assemblies. **(B)** The highly self-assembled liprin-α proteins, indicated by red arrows, organize different receptor clustering and protein condensates (indicated by dotted circles) on both presynaptic and postsynaptic membrane in synapse formation, although no direct evidence showing the involvement of liprin-α in the clustering of Neurexin and the condensation of the GIT1/PIX complex.

Liprin-αs are multiple-domains scaffold proteins mediating various synaptic protein assemblies through both their conserved N-terminal coiled coils and C-terminal SAM123. In these assemblies, each part of the liprin-α protein has its unique role. While SAM123 serves as a tunable hub to accommodate the different binding partners to form the large complexes, the coiled coils undergo regulated self-assembly to control the protein assemblies (Figure 5A).

The self-assembly of liprin-α not only accumulates many synaptic proteins essential for synapse formation, but also organizes these proteins on the presynaptic and postsynaptic membrane to regulate their functions. In addition to the promoting effect on LAR clustering (Xie et al., 2020) and the ELKS condensate formation in presynpase maturation (Liang et al., 2020; McDonald et al., 2020), liprin-αs are involved in several other protein assemblies in the synapse (Figure 5B). Through the interaction with CASK, the key binding partner of Neurexin in the presynapse (Butz et al., 1998; Tabuchi et al., 2002; Dean et al., 2003), liprin-αs may contribute to the presynaptic clustering of Neurexin, which plays a crucial role in the alignment of the presynaptic and postsynaptic machinery during presynaptic differentiation (Dean et al., 2003; Sudhof, 2017). Recently, two members of the LAR-RPTP family, PTPσ and PTPδ were reported to interact with Neurexin to coordinate the presynaptic assemblies (Han et al., 2020). Therefore, liprin-αs may also regulate the Neurexin-mediated assembly via LAR-RPTPs. At the postsynaptic terminal, the self-assembly of liprin-α may enhance the liprin-α/GIT1 interaction that is required for the AMPA receptor clustering (Wyszynski et al., 2002; Im et al., 2003). Finally, as the condensate formation of GIT1 and PIX was proposed to modulate the post-synaptic density (PSD) (Zhu et al., 2020), the self-assembled liprin-α may function as an upstream promoting factor for the regulation of PSD by binding to GIT1.

In support of the master scaffolding role of liprin-α, over 26 proteins have been identified to interact with liprin-αs for diverse functions (Table 1). For instance, liprin-αs assembles ELSK, RIM, and UNC13/Munc13 in the active zone for regulating synaptic vesicle release (Schoch et al., 2002; Deken et al., 2005; Bohme et al., 2016; Dong et al., 2018) and associates with KIF1A to regulate the axonal transport of vesicles (Shin et al., 2003; Hsu et al., 2011; Wu et al., 2016; Zhang et al., 2016; Stucchi et al., 2018). However, due to lacking structural and biochemical information, the molecular basis of these interactions remains elusive. The future structural research of liprin-α-mediated protein interactions will further advance our understanding of how proteins are spatiotemporally orchestrated to control neuron development and synaptic transmission.

## Author Contributions

XX and ML analyzed the structures and prepared the figures. XX and ZW drafted the manuscript. XX, ML, CY, and ZW revised and finalized the manuscript. All the authors contributed to the article and approved the submitted version.

## Conflict of Interest

The authors declare that the research was conducted in the absence of any commercial or financial relationships that could be construed as a potential conflict of interest.
